# Reptile occurrences data in the Volga River basin (Russia)

**DOI:** 10.3897/BDJ.8.e58033

**Published:** 2020-10-30

**Authors:** Andrey Bakiev, Alexander Kirillov, Nadezhda Kirillova, Alexander Ruchin, Anastasia Klenina, Roman Gorelov, Natalya Kostina

**Affiliations:** 1 Samara Federal Research Center of Russian Academy of Sciences, Institute of Ecology of the Volga River basin of Russian Academy of Sciences, Tolyatti, Russia Samara Federal Research Center of Russian Academy of Sciences, Institute of Ecology of the Volga River basin of Russian Academy of Sciences Tolyatti Russia; 2 Mordovia State Nature Reserve and National Park “Smolny”, Saransk, Russia Mordovia State Nature Reserve and National Park “Smolny” Saransk Russia

**Keywords:** Agamidae, Anguidae, Boidae, Colubridae, Emydidae, Gekkonidae, Lacertidae, Lamprophiidae, Reptilia, Viperidae

## Abstract

**Background:**

The Volga basin is one of the most industrially-developed regions of Russia with a high degree of anthropogenic impact on natural ecosystems. Human influence negatively affects the species diversity and number of animals, including reptiles. There are no endemic species in the reptile fauna of the Volga basin. The herpetofauna of the region makes up 25% of the reptile fauna of Russia (Dunaev and Orlova 2017). We began to study the fauna of reptiles and their distribution in the Volga basin in 1988. Although we registered 20 reptile species in the Volga basin to date, apparently this is not a complete list of species in the region (Bakiev et al. 2004, Bakiev et al. 2009a, Bakiev et al. 2015, Kirillov et al. 2020). The distribution of reptiles in this region is not fully understood.

**New information:**

Our dataset contains information on reptile occurrences in the Volga River basin. The dataset is based on original research by the staff of the Laboratory of Herpetology and Toxinology and Laboratory of Population Ecology of the Institute of Ecology of the Volga River basin of the Russian Academy of Sciences and Joint Directorate of the Mordovia State Nature Reserve and National Park “Smolny”. A total of 5,086 occurrences of 20 species are published for the first time with georeferencing. Many of these reptiles are listed in regional Red Data Lists. The European Pond Turtle *Emys orbicularis* (Linnaeus, 1758) is included in the IUCN Red List with the category “Near Threatened”.

## Introduction

The Volga is the longest river in Europe and the 16th largest in the world with 3690 km in length (Litvinov et al. 2009). The area of the Volga basin is about 1.4 million km^2^, which is 33% of European Russia and almost 13% of the territory of Europe. There are different types of biomes in the region, such as taiga in the north and semi-desert in the south (Litvinov et al. 2009). The Volga basin includes all or part of the territory of 29 oblasts, eight republics and one krai of Russia, as well as two oblasts of Kazakhstan (Rosenberg 2009). Previous herpetological studies suggested that more than 20 species of reptiles lived in the Volga basin. Some of the previously-noted species may have been identified incorrectly or have disappeared by now in the Volga basin. For example, I.G. Georgi (1801) mentions "*Testudo caspica*" – *Mauremys caspica* (Gmelin, 1774) – on the Lower Volga. One species – *Tenuidactylus caspius* (Eichwald, 1831) – is considered to have been introduced presumably at the end of the 20th century (Pestov et al. 2009, Andreev et al. 2019).

The first data about the reptiles of the Volga basin appeared in the 10th century (Ibn-Fadlan 1939). However, reliable species identification of the snakes mentioned by Ibn-Fadlan is not possible.

Adam Olshlegel (Olearius 1663, S. 358-359) in 1636 encountered the "Rotbunte" snake – *Natrix tessellata*. The record referred to the left Bank of the Volga in the vicinity of Samara.

The main composition of the herpetofauna of the Volga basin was established by the participants of the 1768-1774 academic expedition: P.S. Pallas (Pallas 1771, Pallas 1776, Pallas 1799, Pallas 1814), I. I. Lepekhin (Lepekhin 1771, Lepekhin 1772), J. P. Falk (Falk 1786, Falk 1824) and J. G. Georgi (Georgi 1775, Georgi 1801, Georgi 1802). P.S. Pallas described two new species of reptiles from the territory of the Volga basin and I. I. Lepekhin described four species.

Specialists from the Institute of Ecology of the Volga River basin of Russian Academy of Sciences began the study of the herpetofauna of the region in 1984, when the Institute was founded (Bakiev et al. 2009, Eplanova and Gorelov 2009, Fayzulin et al. 2009, Klenina et al. 2019, Klenina and Atyasheva 2020, Klenina 2020). In addition, many studies have been conducted in different regions of the Volga basin over the past 20 years. These studies investigated both the ecological and genetic features of species and post-fire changes in herpetofauna (Efimov et al. 2011, Roitberg et al. 2013, Litvinov et al. 2013, Eplanova et al. 2018, Bakiev et al. 2018, Bakiev et al. 2019, Lebedinskii et al. 2019).

The purpose of this paper is to describe a dataset on occurrences of reptiles in the Volga River basin (European Russia) recently published by us in GBIF as a Darwin Core Archive (Kirillov et al. 2020). This paper was prepared according to the concept of “data paper” (Chavan and Penev 2011, Penev et al. 2017).The main idea of the data paper is to publish the usually not easily accessible or discoverable primary biodiversity data as a scientific publication, thus providing free and open access to that information. This is essential for making informed decisions in biodiversity conservation.

## Project description

### Title

Reptile occurrences data in the Volga River basin (Russia).

### Personnel

Alexander Kirillov, Nadezhda Kirillova, Andrey Bakiev, Alexander Ruchin, Anastasiya Klenina, Roman Gorelov, Natalya Kostina.

### Study area description

The Volga River basin is situated in the Russian Plain – centre of European Russia. Water resources of the Volga basin account for only about 5% of the water resources of all Russia. The Volga riverbed and its tributaries are located along the lowlands and only in some places the river passes through the hills (Zhiguli Hills on the Samarskaya Luka). A network of reservoirs has been created on the Volga and its tributaries. There are eight very large reservoirs only on the Volga itself. This determines the configuration of the Volga basin as a whole: below the confluence of the Volga with the Kama, the formation of the lateral inflow practically ends (Rosenberg 2009).

The Volga basin is divided into three regions named as the Upper, Middle and Lower Volga. Gorky and Zhigulevskaya dams are considered the borders of the Upper and Middle Volga and the Middle and Lower Volga, respectively. The Volga basin covers various latitudinal climatic zones. The large extent of the Volga basin from north to south is characterised by a change in biomes from taiga to semi-desert. There is a forest belt on the north of the Volga basin formed by southern taiga and mixed coniferous-deciduous forest. To the south of the forest belt, there is a forest-steppe biome. Biomes of steppes, semi-deserts and deserts are located in the extreme south of the Volga basin. The desert biome is adjacent to the southern part of the Akhtuba River floodplain near the Caspian lowlands (Litvinov et al. 2009).

The climate of the Upper Volga basin is moderate continental, characterised by cold winters and relatively warm summers. The climate of the Middle Volga region is similar to the climate of the Upper Volga region in winter, but the summer climate is less variable. The climate of the Lower Volga region is predominantly continental, characterised by hot summers and relatively short, but cold winters (Litvinov et al. 2009).

### Design description

**Figure 1.**
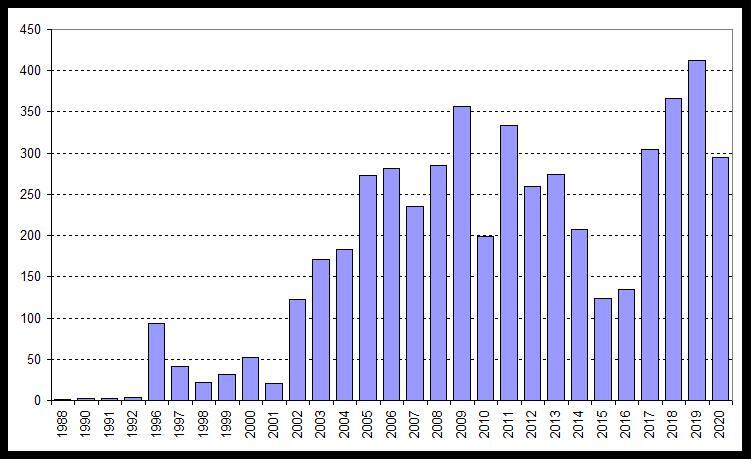
The number of original herpetological studies in the Volga basin (Bakiev and Fayzulin 2002, Bakiev et al. 2009a, Bakiev et al. 2004, Bakiev et al. 2015, Kirillov et al. 2020).

The study of the distribution of reptiles in the Volga basin was carried out during field scientific expeditions by manual capture and species identification of the encountered individuals. Since 2002, the number of original studies of reptiles in the region has increased (Fig. 1). While studying the Volga basin, it was possible to collect data as of common reptile species as rare species, which are listed in the various regional Red Lists.

## Sampling methods

### Sampling description

The dataset is based on personal records from field diaries, some of them having been published earlier, but schematically and without a geographical coordinate reference (Bakiev and Fayzulin 2002, Bakiev et al. 2004, Bakiev et al. 2015). The coordinate reference to each reptile sampling point is given for the first time in this dataset.

### Quality control

Most of the data was collected by herpetologists. The rest of the data was identified by herpetologists from the Institute of Ecology of the Volga River Basin of RAS from photographs we received from amateur naturalists.

### Step description

The fields’ names of the dataset were chosen according to Darwin Core (Wieczorek et al. 2012) and include the following: “occurrenceID”, “basisOfRecord”, scientificName”, “kingdom”, “phylum, “class”, “order”, “family”, “geodeticDatum”, “coordinateUncertaintyInMetres”, “coordinatePrecision” “decimalLatitude”, “decimalLongitude”, “country”, “countryCode”, “individualCount”, “eventDate”, “recordedBy”, “identifiedBy”.

The geographical reference, in most cases, was carried out by determining the coordinates of the finding place of reptiles using a GPS navigator. In other cases, where the geographical coordinates were not obtained using a GPS navigator, but there were original data about the reptile finding places, we determined the coordinates using Google maps (https://www.google.ru/maps/). The margin of error in the measurement of coordinates is 10 m. The accuracy of determining coordinates is up to the fourth digit. In all cases, the WGS-84 coordinate system is used.

## Geographic coverage

### Description

**Figure 2.**
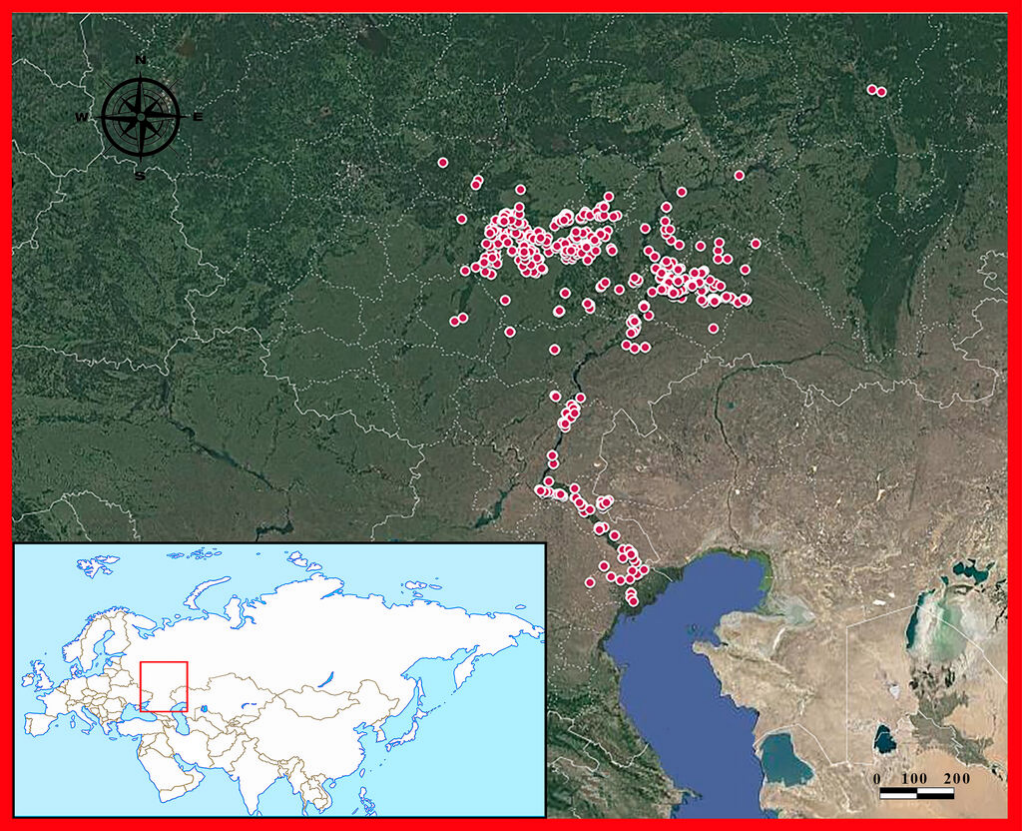
Reptile studying localities within the Volga River basin. The locations of reptile occurrences are shown as red circles. The map of locations was made on Google map by the authors of the article.

The Volga River basin is located in the Russian Plain (Fig. 2). There are the world’s northernmost populations of dice snake *Natrix tessellata* in the Volga basin (Bashkortostan and Samara oblast) (Bakiev et al. 2009a, Yakovlev et al. 2016). Samara oblast also contains Europe's northernmost populations of steppe-runner *Eremias arguta* and the Pallas’ coluber *Elaphe dione* (Eplanova et al. 2001, Bakiev et al. 2004). The Volga basin (Astrakhan oblast) contains the only European population of the even-fingered gecko *Alsophylax pipiens* (Kuzmin and Semenov 2006).

### Coordinates

45°40'48'' and 60°26'49.2'' Latitude; 57°24'21.6'' and 34°8'16.8'' Longitude.

## Taxonomic coverage

### Description

**Figure 3.**
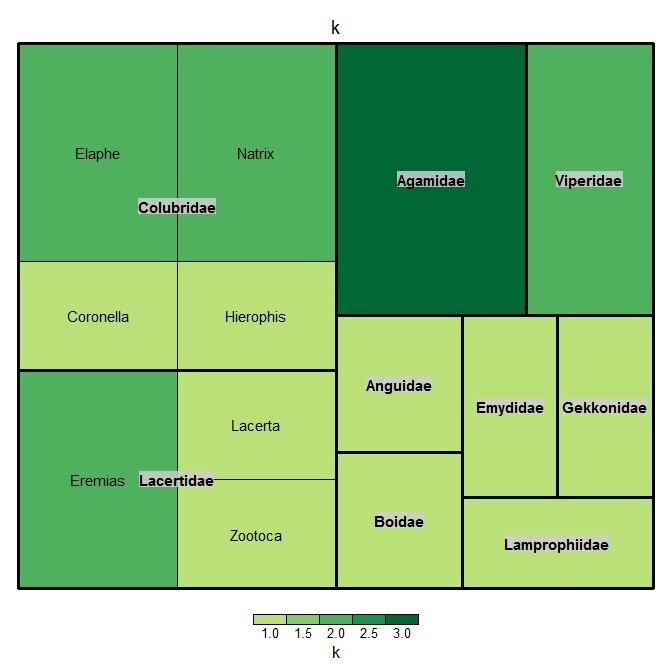
Taxonomic distribution of species amongst reptiles families in the dataset. The figure was prepared with the “treemap” package in R (Tennekes 2017).

All reptile individuals were identified to the species level. The database contains 20 species, belonging to nine reptile families (Fig. 3). Taxonomic affiliation is determined according to the Determinant Atlas of Amphibians and Reptiles of Russia (Dunaev and Orlova 2017), in accordance with the recommendations of the "International Code of Zoological Nomenclature" Ride (1999) and is accepted in this database according to the GBIF.

### Taxa included

**Table:** 

Rank	Scientific Name	Common Name
phylum	Chordata	chordates
class	Reptilia	reptiles
order	Squamata	squamates
order	Testudines	turtles
family	Agamidae	agamids
family	Anguidae	anguids
family	Boidae	boid snakes
family	Colubridae	colubrid
family	Emydidae	pond turtles
family	Gekkonidae	geckos
family	Lacertidae	lacertas
family	Lamprophiidae	lamprophiids
family	Viperidae	vipers
species	*Emys orbicularis* (Linnaeus, 1758)	European pond turtle
species	*Alsophylax pipiens* (Pallas, 1814)	even-fingered gecko
species	*Phrynocephalus guttatus* (Gmelin, 1789)	spotted toadhead agama
species	*Phrynocephalus helioscopus* Pallas, 1771	sunwatcher toadhead agama
species	*Phrynocephalus mystaceus* (Pallas, 1776)	secret toadhead agama
species	*Anguis colchica* (Nordmann, 1840)	slowworm
species	*Eremias arguta* Pallas, 1773	steppe-runner
species	*Eremias velox* Pallas, 1771	rapid racerunner
species	*Lacerta agilis* Linnaeus, 1758	sand lizard
species	*Zootoca vivipara* (Lichtenstein, 1823)	viviparous lizard
species	*Eryx miliaris* (Pallas, 1773)	desert sand boa
species	*Coronella austriaca* Laurenti, 1778	smooth snake
species	*Elaphe dione* (Pallas, 1773)	Pallas’ coluber
species	*Elaphe sauromates* (Pallas, 1814)	Sarmatian rat snake
species	*Hierophis caspius* (Gmelin, 1789)	Caspian whipsnake
species	*Natrix natrix* (Linnaeus, 1758)	grass snake
species	*Natrix tessellata* (Laurenti, 1768)	dice snake
species	*Malpolon monspessulanus* (Hermann, 1804)	Montpellier snake
species	*Vipera berus* (Linnaeus, 1758)	common European viper
species	*Vipera renardi* (Christoph, 1861)	steppe viper

## Traits coverage

### Enter subsection title

Enter subsection text

## Usage licence

### Usage licence

Other

### IP rights notes

Creative Commons Attribution (CC-BY) 4.0 License

## Data resources

### Data package title

Occurrence of the reptiles in the Volga River basin (Russia).

### Resource link

https://www.gbif.org/dataset/2fdc8f80-3f12-482e-ac28-8683d448e881

### Alternative identifiers

https://doi.org/10.15468/n4tztr

### Number of data sets

1

### Data set 1.

#### Data set name

Occurrence of the reptiles in the Volga River basin (Russia).

#### Data format

Darwin Core Archive format

#### Number of columns

19

#### Character set

UTF-8

#### Download URL

https://www.gbif.org/dataset/2fdc8f80-3f12-482e-ac28-8683d448e881

#### Description

Our dataset contains information on reptile occurrences in the Volga River basin, located in the Russian Plain, European Russia. The dataset is based on our own research by the staff of the Laboratory of Herpetology and Toxinology and Laboratory of Population Ecology of the Institute of Ecology of the Volga River basin of the Russian Academy of Sciences and Joint Directorate of the Mordovia State Nature Reserve and National Park “Smolny”. The dataset summarises reptile occurrences noted by field studies in various areas of the Volga River basin from 1988 to 2020. The dataset consists of 5,086 occurrence records, all of them being georeferenced. A total of 20 reptile species belonging to 14 genera and nine families are reported in the Volga River basin, although the distribution of reptile species in this region of Russia has not yet been fully studied.

**Data set 1.** 

Column label	Column description
occurrenceID	An identifier for the Occurrence (as opposed to a particular digital record of the occurrence).
basisOfRecord	Recommended best practice is to use the standard label of one of the Darwin Core classes.
scientificName	The full scientific name, with authorship and date information, if known. When forming part of an Identification, this should be the name in the lowest level taxonomic rank that can be determined. This term should not contain identification qualifications, which should instead be supplied in the IdentificationQualifier term.
kingdom	The full scientific name of the kingdom in which the taxon is classified.
phylum	The full scientific name of the phylum or division in which the taxon is classified.
class	The full scientific name of the class in which the taxon is classified.
order	The full scientific name of the order in which the taxon is classified.
family	The full scientific name of the family in which the taxon is classified.
geodeticDatum	The ellipsoid, geodetic datum or spatial reference system (SRS) upon which the geographic coordinates given in decimalLatitude and decimalLongitude are based.
coordinateUncertaintyInMetres	The horizontal distance (in metres) from the given decimalLatitude and decimalLongitude describing the smallest circle containing the whole of the Location. Leave the value empty if the uncertainty is unknown, cannot be estimated or is not applicable (because there are no coordinates). Zero is not a valid value for this term.
coordinatePrecision	A decimal representation of the precision of the coordinates given in the decimalLatitude and decimalLongitude.
decimalLatitude	The geographic latitude (in decimal degrees, using the spatial reference system given in geodeticDatum) of the geographic centre of a Location. Positive values are north of the Equator, negative values are south of it. Legal values lie between -90 and 90, inclusive.
decimalLongitude	The geographic longitude (in decimal degrees, using the spatial reference system given in geodeticDatum) of the geographic centre of a Location. Positive values are east of the Greenwich Meridian, negative values are west of it. Legal values lie between -180 and 180, inclusive.
country	The name of the country or major administrative unit in which the Location occurs.
countryCode	The standard code for the country in which the Location occurs.
individualCount	The number of individuals represented present at the time of the Occurrence.
eventDate	The date-time or interval during which an Event occurred. For occurrences, this is the date-time when the event was recorded. Not suitable for a time in a geological context.
recordedBy	A person, group or organisation responsible for recording the original Occurrence.
identifiedBy	A list (concatenated and separated) of names of people, groups or organisations who assigned the Taxon to the subject.
